# Fatal Septicemia Linked to Transmission of MRSA Clonal Complex 398 in Hospital and Nursing Home, Denmark

**DOI:** 10.3201/eid2205.151835

**Published:** 2016-05

**Authors:** Rikke Thoft Nielsen, Michael Kemp, Anette Holm, Marianne Nielsine Skov, Mette Detlefsen, Henrik Hasman, Frank Møller Aarestrup, Rolf Sommer Kaas, Jesper Boye Nielsen, Henrik Westh, Hans Jørn Kolmos

**Affiliations:** Author affiliations: Odense University Hospital, Odense, Denmark (R.T. Nielsen, M. Kemp, A. Holm, M.N. Skov, M. Detlefsen, H.J. Kolmos);; Denmark Technical University National Food Institute, Kongens Lyngby, Denmark (H. Hasman, F.M. Aarestrup, R.S. Kaas);; Hvidovre University Hospital, Hvidovre, Denmark (J.B. Nielsen, H. Westh)

**Keywords:** methicillin-resistant Staphylococcus aureus, bacterial infections, bacteremia, disease transmission, infectious, livestock, molecular epidemiology, bacteria, antimicrobial resistance, staphylococci, spa typing

## Abstract

We describe 2 fatal cases of methicillin-resistant *Staphylococcus aureus* (MRSA) clonal complex 398 septicemia in persons who had no contact with livestock. Whole-genome sequencing of the isolated MRSA strains strongly suggest that both were of animal origin and that the patients had been infected through 2 independent person-to-person transmission chains.

Methicillin-resistant *Staphylococcus aureus* (MRSA) clonal complex (CC) 398 is associated with livestock and can spread to humans who have contact with animals (*1*,*2*). The percentage of persons infected with MRSA CC398 is increasing rapidly. In 2014, MRSA CC398 accounted for 43% of all cases of MRSA infection in Denmark (*3*). MRSA CC398 has not been thought to spread easily from person to person (*1*,*2*,*4*) and had been regarded as less virulent than other human MRSA strains (*2*,*5*). Recent studies have showed that MRSA CC398 is an increasing cause of colonization and infection among persons with and without livestock exposure in Germany, the Netherlands, and Denmark (*6*–*8*). However, the transmission route of MRSA CC398 of animal origin to persons with no reported contact with livestock is still unknown. Only a few small outbreaks of MRSA CC398 infection have been reported (*9*–*11*), but animal origin of the bacteria was not documented in those cases.

We describe 2 fatal cases of septicemia attributable to MRSA CC398 of animal origin in a hospital hemodialysis unit and a nursing home. Neither of the patients had any reported contact with livestock. The results of our investigation strongly suggest that transmission occurred through asymptomatic carriers in the 2 institutions.

## The Study

### Transmission Chain 1

#### Patient 1

A 63-year-old man with diabetes and end-stage kidney failure had been receiving maintenance hemodialysis in the outpatient clinic at Odense University Hospital in Odense, Denmark, since 1997. A femoral–femoral bridge graft was used for vascular access. In November 2013, he was admitted to the hospital because of a fever he experienced during dialysis and inflammation around his bridge graft. Cultures of blood samples taken at admission grew MRSA CC398 *spa* type t011. The organism was also cultured from the patient’s bridge graft and from a sample of joint fluid from his right shoulder. A transesophageal echocardiography revealed mitral valve endocarditis. Despite relevant treatment with vancomycin and rifampin and surgical debridement of his shoulder joint, blood cultures remained positive for MRSA until he died 3 weeks later. On inquiry by the staff, the patient had reported no previous history of MRSA infection or colonization and no direct or household-related contact with pigs.

#### Patient 2

Four months before patient 1’s illness, MRSA CC398 *spa* type t011 had been cultivated from an infected decubitus ulcer of another patient who was receiving hemodialysis in the same outpatient clinic as patient 1. Subsequent MRSA screening revealed that patient 2 was a nasal and pharyngeal carrier. On inquiry by the staff, the patient reported no direct or household-related contact with pigs.

### Transmission Chain 2

#### Patient 3

A 74-year-old nursing home resident had hemiparesis and recurrent aspiration pneumonia after an apoplectic insult. In April 2014, he was admitted to the hospital with severe pneumonia. On admission, he had sepsis. Blood cultures grew MRSA CC398 *spa* type t034, and the organism was found in a tracheal aspirate and from the area around a percutaneous gastrostoma tube. Despite relevant treatment with piperacillin/tazobactam, metronidazole, and vancomycin, the patient died from respiratory failure after 1 week. On inquiry by the staff, the patient and his attending daughter reported no direct or household-related contact with pigs.

#### Patient 4

In July 2010, MRSA CC398 *spa* type t034 had been isolated from a bedsore of another patient living in the same nursing home as patient 3. In November 2013, the organism was isolated once more from the patient, this time from an indwelling urine catheter. No contact with pigs were reported on inquiry of the patient and her family. Patients 3 and 4 lived in the same wing of the nursing home and shared common facilities. After hospital staff recognized the transmission chain, all residents in this wing and all attached staff were screened for MRSA, but none tested positive.

All 4 of the affected patients lived in urban areas. None of them had any proximity to pig farms.

The MRSA CC398 isolates identified in the 2 transmission chains were *spa* types t011 and t034, which are common among livestock-associated MRSA strains (*12*). Draft whole-genome sequencing was performed on 7 isolates from the 4 patients in the 2 transmission chains, and results were compared with similar data for CC398-related MRSA and methicillin-sensitive *S. aureus* isolates from Denmark obtained during a previous study of the global dissemination of isolates belonging to this clonal complex (*13*). Sequence files for the individual isolates from patients are available at the European Nucleotide Archive (http://www.ebi.ac.uk/ena; accession no. PRJEB11281). On the basis of these data, a phylogenetic tree was constructed from single-nucleotide polymorphism differences in genome sequences of isolates from the 4 patients and from the isolates obtained during the previous study by using the S0385 complete genome sequence (GenBank accession no. AM990992.1) as reference (Figure). This analysis showed that the patient isolates within each transmission chain were closely related but that no close relation between the 2 chains existed. In addition, the isolates from both chains clustered among isolates previously found to be animal associated (clade IIa; *13*). Consistent with an animal origin, all isolates carried the *tet*(M) and *czrC* determinants (similar to most MRSA strains from clade IIa) and lacked the ɸSa3 phage (typical of the human clade I) (*13*).

**Figure:**
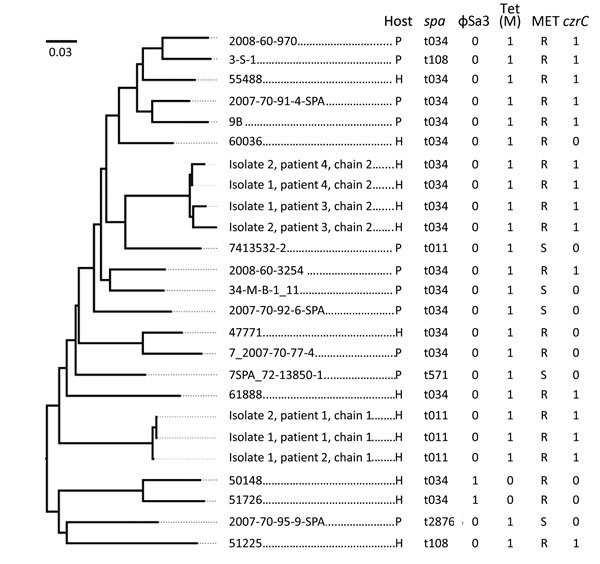
Phylogeny of methicillin-resistant *Staphylococcus aureus* (MRSA) clonal complex (CC) 398 isolates linked to fatal septicemia in a hospital patient and a nursing home resident in Denmark. Draft whole-genome sequencing was performed on 7 isolates from the 4 patients identified in the 2 transmission chains, and results were compared with similar genomic data for CC398-related MRSA and methicillin-sensitive *S. aureus* isolates obtained in Denmark during a previous study of isolates belonging to CC398 (*13*). Single-nucleotide polymorphism differences were identified, and a maximum-likelihood phylogeny was inferred from raw data by using the web tool CSI Phylogeny (https://cge.cbs.dtu.dk//services/CSIPhylogeny). The reference strain was S0385 (GenBank accession no. AM990992.1). The region of bp 12252–135180 was excluded from analysis because it contains the *spa* region and disrupts the phylogenic signal (*13*). Scale bar indicates substitutions per site. P, pig; H, human; MET, methicillin susceptibility; R, resistant; S, susceptible.

## Conclusions

We report 2 fatal MRSA CC398 infections after human-to-human transmission in institutional settings. Both patients had debilitating underlying diseases but were in a stable condition until the time of their infections. The sequence of events leaves no doubt that septicemia attributable to MRSA CC398 was the cause of death in both cases. The CC and *spa* types of the isolates causing fatal infections were typical for MRSA isolates from pigs. Phylogenetic analyses of whole-genome sequences indicated that the human isolates from the 2 transmission chains were located in different clusters that intermingled with isolates from pigs. The detection of molecular markers associated with livestock origin further confirmed animal origin. The different *spa* types and the clustering of the MRSA isolates from the 2 chains clearly indicate 2 separate chains of infection.

None of the 4 patients described here had occupational or household contact with livestock. However, epidemiologic investigations and typing analyses strongly suggested that the 2 MRSA-infected patients could have acquired their infections from an asymptomatic carrier in the outpatient hemodialysis unit (transmission chain 1, patient 2) and the nursing home (transmission chain 2, patient 4). Transmission of MRSA CC398 in hospitals and institutions has been reported elsewhere, which underscores its potential to spread through person-to-person contact (*9*–*11*).

In conclusion, the organism implicated in these 2 fatal cases was by all accounts spread from person to person. These findings suggest that this clonal complex can be of high pathogenicity and is readily transmissible among humans.
